# Enhancing nursing practice through patient outcome measures: a framework for optimizing care in intracranial surgery

**DOI:** 10.1186/s12912-025-02960-x

**Published:** 2025-04-10

**Authors:** Rasha Hassan Abbas Shady, Rasha Hafez Ramadan El-Shaboury, Reham AbdElhamed AbdElmawla Elsaid, Shereen Abd El-Moniem Ahmed, Ghada Gamal Badawy, Warda Elshahat Hamed, Rania Rabie El-Etreby, Ayman Muhammad Kamel Senosy

**Affiliations:** 1 Medical-Surgical Nursing, Faculty of Nursing, Mansoura University, Mansoura, Egypt; 2 Medical-Surgical Nursing, Suez Canal University, Suez, Egypt; 3 Pediatric Nursing, Faculty of Nursing, Mansoura University, Mansoura, Egypt; 4 Psychiatric and Mental Health Nursing, Faculty of Nursing, Mansoura University, Mansoura, Egypt; 5 Medical-Surgical Nursing, Faculty of Nursing, Ain Shams University, Cairo, Egypt

**Keywords:** Nursing practice, Outcome measures, Intracranial surgeries

## Abstract

**Background:**

The nursing practices are evidence-based and have not been systematically applied to patients undergoing intracranial surgeries, which is one of the most critical procedures for treating brain diseases. Nursing care prevents complications, minimizes infections, and ensures a healthy, speedy recovery across preoperative, intraoperative, and postoperative phases.

**Aim:**

This study aimed to evaluate the enhancement of nursing practice through patient outcome measures for optimizing care in intracranial surgeries.

**Method:**

A quasi-experimental design was utilized in this study. The study was conducted at the neurosurgery unit at the Mansoura University Hospital with 50 nurses and 100 patients, 50 for each control and intervention group. The patients were divided into study groups in which the trained nurses applied to nursing practices, and the control group received only their usual care.

**Tools:**

Four tools were used, including a structured interview questionnaire sheet. Nurses’ knowledge and nursing practices for patients undergoing intracranial surgeries, patients’ outcomes, and observational performance checklist.

**Statistical analysis:**

A one-way analysis of variance (ANOVA) test, which is a statistical test used to analyze the difference between the means of more than two groups. The chi-square, SD (standard deviation), MH (marginal homogeneity test), and t (paired t-test) have been involved in the study.

**Results:**

Nurses’ knowledge improved from 42.9% fair pre-application to 54% good post-application (*P* < 0.001). Pre- and postoperative practices also showed marked progress, with satisfactory practice levels rising from 66% to 72% in second observations, respectively (*P* < 0.001). Patient outcomes improved, with 62% of the study group showing moderate positive outcomes compared to 92% negative in the control group (*P* < 0.001). Patient satisfaction scores rose significantly, from a mean of 25.3 pre-application to 83.9 post-application (*P* < 0.001).

**Conclusion:**

The study demonstrated significant improvements in nurses’ knowledge, practices, and patient outcomes following the implementation of the nursing practices.

**Recommendations:**

Implement the nursing practices as a standard protocol to enhance nursing practices, improve patient recovery, and reduce complications in neurosurgical care.

## Introduction

Intracranial disorders are severe health conditions that cause human suffering and impose significant economic burdens. They are also among the most various diseases, with poorly understood underlying mechanisms, which poses a significant challenge to developing effective treatments Maas et al. [1]. Neurosurgical procedures for intracranial disorders, such as burr holes, craniotomy, and craniectomy, are commonly performed in clinical practice to treat these conditions. The primary goal of these procedures is to improve patients’ physical, mental, and emotional well-being. However, the postoperative period remains the most vulnerable and critical phase of the patient’s recovery García et al. [2].

Neuroscience nursing is a specialized area within nursing that focuses on caring for individuals with biopsychosocial disruptions resulting from nervous system dysfunction. This specialty encompasses all levels of human functioning, from basic physiological processes like mobility and sensation to advanced cognitive processes like communication and consciousness. Neuroscience nurses address the needs of patients by diagnosing and managing responses to nervous system impairments. Their care extends beyond patients, including families and caregivers, who often require education and emotional support during recovery Huang et al. [3].

Nursing care for patients undergoing intracranial surgeries can be divided into three essential phases: immediate post-anesthetic, intermediate hospitalization, and convalescent. Each phase demands careful management to maintain homeostasis, alleviate pain, prevent complications, and ensure smooth transitions from the hospital to full recovery. Effective nursing interventions focus on the patient’s physical, psychological, and emotional needs throughout these phases Ahmed et al. [4]. The nursing practices provide a structured framework for organizing nursing care during preoperative and postoperative stages to improve patient outcomes. This ensures quality care by focusing on structure, process, and outcome measures Sharaf Eldeen et al. [5].

Key outcome indicators include the prevention of postoperative complications, stabilization of neurological status, effective management of seizures, and improvement in patient satisfaction. Patient-centered outcomes, such as emotional well-being and anxiety reduction, are equally critical in determining the success of nursing interventions (Algburi et al. [6]), and it is important to clarify the role of psychiatric nursing as part of the patient care plan. Educating and engaging patients in their care is essential for better outcomes. Informed patients who understand their treatment plan and can recognize early warning signs are more likely to prevent complications and adhere to postoperative instructions (El Ashery et al. [7]). As a result, nursing care incorporating patient education and communication strategies is vital for ensuring positive outcomes Lee [8].

Psychiatric nursing is integral to the Nursing Practices (NP) for patients undergoing intracranial surgeries, addressing essential mental and emotional needs that complement physical recovery. Patients frequently experience anxiety, depression, and cognitive changes post-surgery, which can impact overall outcomes. Psychiatric nurses provide mental health assessments, psychological support, and coping strategies to help manage these challenges, enhancing resilience and promoting recovery Cusack et al. [9].

Additionally, they educate patients and families on potential behavioral changes, collaborating closely with neurosurgical teams to support cognitive and emotional rehabilitation Lindlöf et al. [10]. Including psychiatric nursing within the NP supports a holistic, interdisciplinary approach, ensuring comprehensive care for neurosurgical patients Lindlöf et al. & Institute of Medicine (US) [10, 11].

Nursing leaders in professional groups, as well as some government organizations like the Public Health Service’s Bureau of Health Professions, have developed standards and criteria that serve as guidelines for nursing practice. Systematic nursing practices frameworks assist nurses in actual performance. There is widely accepted nursing practice framework such as used in the *nursing process*. Three roles of nurses as investigator, educator, and advocate, make up a paradigm developed by the California Public Health Foundation (CPHF, 1992) to direct medical and nursing practice specifically related to environmental health concerns Coco et al. [12].

Although Nursing Practices have been widely used in several countries since the 1980s and 1990s, it is very vital for optimizing nursing care for patients, there were use lack of widespread adoption highlights a gap in standardized care for patients undergoing intracranial surgeries before in Egypt Duffield [13] which indicats the importance of following good and standardized nursing practices and care for patients post operatively with major surgeries.

According to the Mansoura University Hospital Neurosurgery Department’s 2023 report, 510 patients underwent intracranial surgeries in 2022, with an additional 309 patients on the waiting list for surgical procedures in 2023. Given the increasing demand for neurosurgical care, there is an urgent need to implement and evaluate the enhancing of care quality and patient outcome measures for optimizing care in intracranial surgeries in the neurosurgery unit of Mansoura University Hospital. By identifying gaps and measuring improvements in nursing performance and patient outcomes, the findings of this study are expected to contribute to the standardization of nursing care for intracranial surgery patients and encourage further research in this area.

### Research questions

1. How does the Nursing Practice (NP) impact nurses’ preoperative, intraoperative, and postoperative care knowledge and practices?
2. What are the differences in nursing knowledge and practice levels before and after implementing the NP?
3. To what extent does the implementation of the NP improve patient outcomes, including recovery rates, complication rates, and satisfaction levels?

### Research hypotheses

#### H1

Nurses’ knowledge of preoperative and postoperative care for patients undergoing intracranial surgeries will be improved after implementing the Nursing Practices (NP).

#### H2

Nurses’ clinical practices in caring for intracranial surgery patients will be improved after implementing the Nursing Practices (NP).

#### H3

Patient outcomes will be improved after applying the Nursing Practices.

## Methods

### Research design and setting

A quasi-experimental design with one group pretest-post test type was used in this study to assess the impact of a nursing practice (NP) on patient outcome measures and nurse performance for patients undergoing intracranial surgeries. The study was conducted at the Neurosurgery Unit of Mansoura University Hospital in the Delta region of Egypt. Neurosurgical procedures are performed regularly in this unit, with an average of eight intracranial surgeries conducted weekly.

### Subjects

The study population comprised all available nurses (*n* = 50) working in the Neurosurgery Unit of Mansoura University Hospital and 100 patients. These nurses were directly involved in the care of patients undergoing intracranial surgeries.

### Sample size

Based on data from the literature by Sharaf Eldeen et al. [5], considering a level of significance of 5% and power of study of 80%, the sample size can be calculated using the following formula:

$$\:\text{n}=\frac{2(\text{Z}{\upalpha\:}/2\:+\:\text{Z}{\upbeta\:})^2\:\times\:\:\text{p}\:(1-\text{p})}{\left(\text{d}\right)^2}$$

where, p = pooled proportion obtained from previous study; d = expected difference in proportion of events; Z_α/2_ =1.96 (for 5% level of significance) and Z_β_ = 0.84 (for 80% power of study). Therefore,

$$\:\text{n}=\frac{2{\left(1.96\:+\:0.84\right)}^{2}\times\:\:0.926\:(1-0.926)}{\left(0.148\right)^2}=49.1.$$

Accordingly, the sample size required is 50 in each group.

### Tools of the study

The researchers used four tools for data collection as the following:

#### Tool I: A structured questionnaire

The researchers developed this questionnaire based on a review of relevant scientific literature by El Ashery et al.; Doyle at al & Mostafa et al. [7, 14, 15]. It was used to gather demographic and professional data from the participating nurses, including age, gender, educational level, years of experience, and prior training related to the care of intracranial surgery patients. The questionnaire was written in simple Arabic for clarity and ease of understanding.

#### Tool II: Nurses’ knowledge regarding nursing practice for patients undergoing intracranial surgeries

The researchers developed this tool to assess nurses’ knowledge of patients undergoing intracranial surgeries. It includes both open and closed-ended questions covering several critical areas, such as the meaning of brain surgeries, preoperative and postoperative precautions, and perioperative care practices. Additionally, the tool evaluates the nurse’s ability to identify warning signs and complications that require reporting to the physician and knowledge of seizure management strategies. It also assesses understanding of prescribed medication management, including administration and side effects, along with dietary recommendations essential for recovery and infection control measures. Each correct response is assigned one point, and the total score is converted into a percentage. Nurses who achieve 60% or higher are classified as having “satisfactory” knowledge, while those scoring below 60% are categorized as having “unsatisfactory” knowledge. The scoring and content design are based on the guidelines provided by Doyle at al., and Mostafa et al. [14, 15].

#### Tool III: Postoperative brain surgery patients’ expected outcomes assessment

This tool, which includes two parts, seems to be a comprehensive way to assess the outcomes and satisfaction of postoperative brain surgery patients.

##### Part I: postoperative outcomes assessment

is a valid and reliable standardized tool to measure the absence or decreased post-operative complications, good neurological status, the ability of the caregivers to deal with post-operative seizures, stability of vital signs, stability of laboratory investigations, and reduction of anxiety level Louie et al. [16]. Each achieved expected outcome is scored as one, then numbers converted to percentages with the total score conversion is:

- Adverse outcomes: 50–64%
- Moderate level of positive outcomes: 65–84%
- Positive outcomes: 85–100%

##### Part II: patient satisfaction assessment developed

by Kokou et al. [17]; this part consists of 20 items grouped into five dimensions (assurance, empathy, reliability, responsiveness, tangibility), with four statements per dimension. Responsiveness such as giving attention to patients, responding to questions, while empathy includes communication and consumer access. Each item is scored as one; then numbers converted to percentages with the total score conversion is:

- Lowest level of satisfaction: 33–55%
- Moderate level of satisfaction: 55–77%
- Highest level of satisfaction: 77–100%

This dual-part tool allows for a detailed evaluation of clinical outcomes and patient satisfaction, which can be crucial for improving care quality.

#### Tool IV: Observational performance checklist for applying nursing practices for patients undergoing intracranial surgeries

Hinkle et al. [18]. This tool was adapted from Hamilton Health Science (2019), to be suitable for the study subject and their characteristics. It is designed to assess nurses’ performance in delivering care to patients undergoing intracranial surgeries. It consists of two parts:

##### Part one: preoperative nursing practices

This includes taking a complete patient history (both subjective and objective data), informing and educating the patient about the surgery and its expectations, answering patient questions, obtaining surgical consent, conducting lab investigations, and performing neurological and headache assessments. Additional tasks involve preparing the surgical site, instructing the patient to fast from midnight before the surgery, and administering preoperative medications.

##### Part two: postoperative nursing practices

This includes a checklist of nurses’ activities during recovery, such as wound care, management of wound drains, perioperative monitoring, pain management, and seizure management. Nurses are also evaluated on their ability to implement infection control measures and provide patient education on lifestyle modifications for recovery, such as diet, physical activity, and medication compliance. The checklist ensures that the researcher observes and records each step to assess nursing performance accurately.

##### Scoring system

Each Nursing Practices was evaluated based on competency, with three possible scores: “done adequately” (2 points), “done inadequately” (1 point), or “not done” (0 points). The overall performance of nurses is categorized based on the total score as follows:

- Good Practice: 75–100%
- Satisfactory Practice: 50–74%
- Poor Practice: Less than 50%

### Data collection procedure

Data collection covered 8 months from the beginning of March 2023 to the end of October 2023 (2 months for the preparatory and administrative phase and 6 months for the exploratory phase). Data were collected during morning, afternoon, and night shifts. The fieldwork was carried out throughout four phases to achieve the purpose of the current study as the following:

### Preparatory phase

This phase encompassed obtaining approval to conduct the study, reviewing relevant literature, developing study instruments, and evaluating the tools for reliability and content validity.

### Administrative phase

Following the approval of the study objectives and methods, ethical clearance was obtained from the Faculty of Nursing’s Research Ethical Committee and administrative permission from Mansoura University Hospital. A thorough review of relevant literature was conducted to inform the development of the Nursing Practices (NP) and its study tools. The tools, including questionnaires, checklists, and educational materials, were validated by experts in neurosurgery and nursing to ensure clarity, relevance, and alignment with evidence-based practices.

### Validity and reliability

A panel of ten professional experts from neurosurgery, medical-surgical nursing, and medical biostatistics reviewed the study tools to evaluate their content validity for relevancy, clarity, simplicity, comprehensiveness, and applicability. According to Halek et al. [19], ten experts were enough to offer feedback for content validity. The researchers made all the suggested modifications, and the final tool’s format was prepared. The study tools’ internal consistency was assessed using Cronbach’s coefficient alpha. This technique indicated the high reliability of the final format of the tools. The correlation coefficient was Tool II (r) = 0.93, Tool III (r) = 0.84, and Tool IV (r) = 0.89.

### The exploratory phase

This phase included the pilot study and the fieldwork:

#### Pilot study

The pilot study was conducted on 10% of the total sample (*n* = 5 nurses) to assess the study tools’ clarity, feasibility, and applicability. The participants in the pilot study were excluded from the final sample. Based on the pilot results, minor modifications were made, including adding questions related to wound care in Tool IV.

### The fieldwork was implemented in the following four phases

#### Measurement phase

All study tools were utilized to ensure that the participating nurses and patients met the research’s inclusion criteria, which include that patients would not be exposed to the designed clinical practice protocol. Furthermore, the nurses and patients want to complete participating in the study without the need for withdrawal. Nurses were assessed using structured questionnaires and observational checklists, while patients were screened to confirm their eligibility, which means all patients underwent cranial surgery and based on the study’s defined criteria, which included that patients are free from psychiatric or mental disorders and that the patients have stability and continuity to complete participation in the study.

#### Planning phase

The Nursing Practices (NP) was developed using a one-group pretest-posttest design. All participating nurses underwent a pre-intervention assessment using structured questionnaires and observational checklists to evaluate their baseline knowledge and practices. Based on the preliminary assessment data, nurses’ expectations, and recent literature review of Lv K et al. [20], the researchers designed the NP to address gaps in knowledge and performance. The development of the NP followed a structured “step-by-step guide,” integrating evidence-based practices to ensure its relevance and effectiveness. That was delivered in a simple Arabic booklet containing theoretical content and practical procedures, which was reviewed and validated by specialized professors to ensure clarity and applicability. The patients were divided into study groups in which the trained nurses applied NP, and the control group received only their usual care.

#### Implementation of the “nursing practices” phase

The implementation phase focused on delivering the Nursing Practices (NP) to nurses through three structured sessions. Each session lasted 30 to 45 min, three days per week, during their working hours and it was conducted face-to-face, utilizing a combination of lectures and group discussions to enhance engagement and comprehension. To support learning, each nurse received a printed Arabic booklet containing the theoretical and procedural content of the NP. This resource was a reference to reinforce the knowledge and practices covered during the sessions. Nurses were informed of the schedule for subsequent sessions to ensure full participation.

Each session began with a summary of the previous session’s content and a clear outline of the objectives for the current session. At the end of each session, the researchers summarized the material covered and collected feedback from the nurses to assess their understanding and address any questions.

The training sessions were conducted in small groups of 3 to 5 nurses per session, ensuring personalized attention and active participation. The NP content was designed to provide practical guidance tailored to the care of patients undergoing intracranial surgeries, promoting better knowledge retention and application in clinical practice.

### The nursing practices content was covered in three sessions as follows

#### The first session: (General educational session)

At the beginning of this session, the researcher introduced herself to the nurses and explained important items, such as the general and specific objectives of the NP, schedule, information about the structure and function of the brain, brain surgery, medical treatment after brain surgery, early detection and reporting of postoperative complications, care of the wound, and signs and symptoms of wound infection. (Time allowed: 40–45 min).

#### The second session: (Preoperative Preparation session)

This session comprised routine preoperative care that included immediate preoperative care such as removing jewelry, personal hygiene, assisting the patient with personal hygiene and related care such as bathing or showering, etc.…. as well as neurological assessment and measures to prevent increased intracranial pressure after the operation, wound incision care, as well as demonstrate pursed lip breathing exercises (Time allowed: 40–45 min).

#### The third session included a post-operative care session

This session comprised care of a wound drain, pain management that included pharmacological and non-pharmacological methods, lifestyle changes such as smoking cessation, diet, driving, sexual activity, and weight control, as well as monitoring of blood glucose levels and blood pressure, and activities such as exercises and medication compliance as anticonvulsant and corticosteroid medications and their nursing role, as well as follow-up after discharge to measure outcomes. (Time allowed: 40–45 min).

#### Evaluation phase

After implementing the NP, nurse knowledge, practices, and measure outcomes were reassessed using tools (II, III, and IV) to evaluate patients’ measure outcomes as determinants for utilizing the Nursing Practices for Patients undergoing Intracranial Surgeries. There were direct observations by evaluators.

### Statistical analysis

All statistical analyses were performed using SPSS for Windows version 20.0 (SPSS, Chicago, IL). Continuous data were normally distributed and were expressed in mean ± standard deviation (SD). Categorical data were expressed in numbers and percentages. A one-way analysis of variance (ANOVA) test, which is a statistical test used to analyze the difference between the means of more than two groups. It was used to compare more than two variables with continuous data. The chi-square test that refers to the categorical variable distribution in a sample often requires being compared with the distribution of a categorical variable in another sample; it was used to compare variables with categorical data. The standard deviation (SD), marginal homogeneity test (MH), and t (paired t-test) have been involved in the study. The reliability (internal consistency) test for the questionnaires used in the study was calculated. Statistical significance was set at *p* < 0.05.

## Results

Table 1 displays the percentage distribution of the nurses studied based on their socio-demographic characteristics. The findings showed that more than half of the studied nurses were females (52%), and 56% were older than 30, and the mean ± SD was 34.2 ± 10.3. Less than one-third (30.0%) had completed postgraduate education, and (32%) had more than ten years of experience in the field.

**Table 1:** Percentage distribution of the studied nurses based on their socio-demographic characteristics

	Socio-demographic data	
	*N* = 50	%
**Age**		
< 25	11	22.0%
25–30	11	22.0%
> 30	28	56.0%
**Mean ± SD**	**34.2 ± 10.3**	
**Gender**		
Male	24	48.0%
Female	26	52.0%
**Level of Education**		
Diploma	12	24.0%
Technical nursing	10	20.0%
Bachelor’s degree	13	26.0%
Postgraduate	15	30.0%
**Years of experience in filed**		
< 2	9	18.0%
2 < 5	14	28.0%
5 < 10	11	22.0%
≥ 10	16	32.0%

Table 2 shows the demographic data of the patients’ study population. Regarding age, it shows nearly half, 48% of the control group and 46% of the study group, were more than 50 years old, with mean ages of 48.2 ± 7.7 and 48.6 ± 7.6, respectively. Regarding gender, males dominated both groups, 64% and 58%, respectively. Furthermore, 48% and 50% in both groups had previous intracranial surgery. Regarding patient complaints, 28% and 24% of both groups reported feeling dizziness, 24% and 22% reported having seizures, respectively, and 20% and 18% reported experiencing a change in consciousness in that order.

**Table 2:** The demographic data of the patient’s study population

Demographic data	Control group		Study group		Significance	
	*n* = 50	%	*n* = 50	%	X^2^/t	*P*
**Age**						
< 40	9	18.0	7	14.0	0.515	0.773
40–50	17	34.0	20	40.0		
> 50	24	48.0	23	46.0		
**Mean ± SD**	48.2 ± 7.7		48.6 ± 7.6		0.237	0.813
**Gender**						
Female	18	36.0	21	42.0	0.378	0.539
Male	32	64.0	29	58.0		
**Previous Intracranial surgery**	24	48.0	25	50.0	0.040	0.841
**Complaints**						
Headache	8	16.0	9	18.0	0.909	0.923
Change in consciousness	10	20.0	9	18.0		
Visual disturbances	6	12.0	9	18.0		
Dizziness	14	28.0	12	24.0		
Seizures	12	24.0	11	22.0		
**Smoking**	21	42.0	24	48.0	0.364	0.546
**Radiological Examination**						
CT	19	38.0	22	44.0	0.372	0.542
MRI	31	62.0	28	56.0		

**P**: P- value for comparing between control and study groups --*: Statistically significant at *p* ≤ 0.05

Table 3 indicates that the overall nurses’ knowledge score at the pre-and post-application NP differed statistically significantly. Most nurses gained good knowledge post-application of NP regarding pre- and postoperative precautions, warning signs, seizure(s), prescribed medicine, infections, and intracranial pressure (*P* = 0.001).

**Table 3:** Studied nurses’ knowledge score and test of significance in relation to overall knowledge pre and post application of nursing practices for patients undergoing intracranial surgeries

Knowledge	Pre		Post		X^2^	*P*
	*n* = 50	%	*n* = 50	%		
The meaning of intracranial surgery	26	52.0	36	72.0	4.244	0.039
Pre and postoperative precautions	22	44.0	38	76.0	10.667	< 0.001
Warning signs	19	38.0	42	84.0	22.236	< 0.001
How to deal with seizure(s)	23	46.0	36	72.0	6.986	0.008
Prescribed Medicine	18	36.0	39	78.0	17.993	< 0.001
Dietary measures	22	44.0	35	70.0	6.895	0.009
Infections	17	34.0	40	80.0	21.583	< 0.001
Bleeding	16	32.0	37	74.0	17.704	< 0.001
Intracranial Pressure	22	44.0	37	74.0	9.301	0.002
Wound care	24	48.0	34	68.0	4.105	0.043

**P**: P value for comparing between pre and post -- *: Statistically significant at *p* ≤ 0.05

Table 4 displays the nurse’s studied knowledge score and test of significance about overall knowledge pre- and post-application of the Nursing Practices. The findings showed that more than half of the nurses studied (54.0%) gained good knowledge scores post-application of the Nursing Practices compared with (42.9%) of those who had fair knowledge scores pre-implementation of the Nursing Practices (NP). The results revealed a significant difference (P value < 0.001) in nurses’ knowledge level post-application of nursing practices.

**Table 4:** Studied Nurse’s knowledge score and test of significance in relation to overall knowledge pre and post implementation of nursing practices

Total knowledge score	Pre		Post		Test of Sig.	*p*
	*n* = 50	%	*n* = 50	%		
Poor knowledge	**25**	51.0	**3**	6.0	**MH=36.504**	**< 0.001** ^*^
Fair knowledge	**21**	42.9	**20**	40.0		
Good knowledge	**3**	6.1	**27**	54.0		
Mean ± SD	**4.2 ± 2.1**		**7.5 ± 1.6**		**8.834**	< 0.001*

SD: Standard deviation MH: Marginal Homogeneity Test t: Paired t-test

**P**: P value for comparing between pre and post -- *: Statistically significant at *p* ≤ 0.05

Table 5: There was a difference between the first and second observation periods regarding nurses’ preoperative practices. More than two-thirds of nurses (78%) had poor practice levels in the first observation, while almost two-thirds (66%) had satisfactory practice levels. The mean score for preoperative practice was 7.5 ± 2.3 in the first observation and 12.5 ± 2.7 in the second observation. There was a statistically significant difference in preoperative nursing practices between the first and second observation periods (P value < 0.001).

**Table 5:** Comparison of Pre-Operative nursing practice improvements across two observational periods

	1st Observation						2nd Observation						X^2^	*P*
	Not done		Done inadequately		Done adequately		Not done		Done inadequately		Done adequately			
	*n* = 50	%	*n* = 50	%	*n* = 50	%	*n* = 50	%	*n* = 50	%	*n* = 50	%		
Taking complete History	27	54	12	24	11	22	11	22	16	32	23	46	11.544	0.003
Informing and instructing the patient about the surgery and its expectations	20	40	17	34	13	26	11	22	14	28	25	50	6.693	0.035
Providing answers for patient questions	25	50	17	34	8	16	10	20	15	30	25	50	15.311	< 0.001
Surgical consent	22	44	16	32	12	24	13	26	13	26	24	48	6.625	0.036
Lab investigations	24	48	17	34	9	18	15	30	15	30	20	40	6.374	0.041
Neurological assessment	26	52	12	24	12	24	13	26	15	30	22	44	7.608	0.022
Headache assessment	26	52	11	22	13	26	9	18	16	32	25	50	12.973	0.002
Preparation of surgical site	20	40	19	38	11	22	13	26	14	28	23	**46**	6.478	0.039
Instructing patient to fast form midnight at the day of the operation,	25	50	14	28	11	22	9	18	19	38	22	44	11.954	0.003
Administration of pre-operative medication	20	40	20	40	10	20	7	14	18	36	25	50	12.793	0.002

Total Practice	Poor		Satisfactory		Good		Poor		Satisfactory		Good			
	*n* = 50	%	*n* = 50	%	*n* = 50	%	*n* = 50	%	*n* = 50	%	*n* = 50	%		
Total preoperative practice level	39	78	11	22	0	0	5	10	33	66	12	24	49.273	< 0.001
Total preoperative practice mean score	7.5 ± 2.3						12.5 ± 2.7						9.868	< 0.001

*: Statistically significant at *p* ≤ 0.05

Table 6 demonstrates a comparison of the postoperative nursing practices between the first and second observation periods; regarding total postoperative practice level, it was noticed that the majority, 90% of nurses, had poor practice levels in the first observation and more than two-thirds, 72%, of nurses had satisfactory practice levels in the second observation. Finally, the total practice mean score was 5.2 ± 1.8 in the first observation and 9.1 ± 1.9 in the second, with a statistically significant difference in nursing practices between the first and second observation periods (P value < 0.001).

**Table 6:** Comparison of the post-operative nursing practices between the 1st and 2nd observation periods

Items	1st Observation						2nd Observation						X^2^	*P*
	Not done		Done inadequately		Done adequately		Not done		Done inadequately		Done adequately			
	*n* = 50	%	*n* = 50	%	*n* = 50	%	*n* = 50	%	*n* = 50	%	*n* = 50	%		
Patient assessment	26	52	12	24	12	24	10	20	19	38	21	42	11.146	0.004
Care of wound site	25	50	18	36	7	14	8	16	19	38	23	46	17.318	< 0.001
Care of wound drain	20	40	17	34	13	26	5	10	14	28	31	62	16.654	< 0.001
Perioperative care	22	44	13	26	15	30	10	20	21	42	19	38	6.853	0.033
Pain management measures	24	48	14	28	12	24	8	16	15	30	27	54	13.804	< 0.001
How to deal with post-operative seizures	22	44	20	40	8	16	10	20	18	36	22	44	11.139	0.004
Infection control measures	25	50	16	32	9	18	10	20	16	32	24	48	13.247	< 0.001
Providing health teaching for patient about lifestyle modifications	22	44	14	28	14	28	10	20	19	38	21	42	6.658	0.036

Total postoperative Practice	Poor		Satisfactory		Good		Poor		Satisfactory		Good			
	*n* = 50	%	*n* = 50	%	*n* = 50	%	*n* = 50	%	*n* = 50	%	*n* = 50	%		
Total postoperative practice level	45	90	5	10	0	0	9	18	36	72	5	10	52.43	< 0.001
Total postoperative practice mean score	5.2 ± 1.8						9.1 ± 1.9						10.28	< 0.001

*: Statistically significant at *p* ≤ 0.05

Table 7: Most of the subjects in the control group (92%) demonstrated adverse outcomes, while more than half of the study group (62%) demonstrated moderate positive outcomes, with statistically significant differences revealed between the two groups regarding “measures outcomes,” where p (< 0.001).

**Table 7:** Comparison between two groups of patients (control & study) to measure patients’ outcomes post applying the nurses nursing practices studied

Items	Control				Study				Chi square test	
	Not Achieved		Achieved		Not Achieved		Achieved			
	*n* = 50	%	*n* = 50	%	*n* = 50	%	*n* = 50	%	X^2^	*P*
Reports headache is relieved or decreased	34	68	16	32	18	36	32	64	10.256	< 0.001
Patient and/or a family member report how to deal with patient if seizure occur	32	64	18	36	17	34	33	66	9.004	0.003
Demonstrates use of therapeutic interventions (e.g., relaxation skills) to relieve pain	35	70	15	30	16	32	34	68	14.446	< 0.001
Demonstrates stabilized vital signs	34	68	16	32	17	34	33	66	11.565	< 0.001
Demonstrates stabilized laboratory investigations	34	68	16	32	15	30	35	70	17.647	< 0.001
Verbalizes understanding of condition, prognosis	33	66	17	34	16	32	34	68	11.565	< 0.001
List signs/symptoms requiring medical follow-up	34	68	16	32	13	26	37	74	17.704	< 0.001
Initiates Diet	30	60	20	40	11	22	39	78	14.924	< 0.001
Initiates Exercise	31	62	19	38	15	30	35	70	10.306	< 0.001
Reduce smoking	33	66	17	34	16	32	34	68	11.565	< 0.001
Recognizes need for seeking assistance with some activity as his condition as needed	34	68	16	32	13	26	37	74	17.704	< 0.001
Demonstrates satisfaction with the educational Program	32	64	18	36	18	36	32	64	16.234	< 0.001
**Post-operative patients’ total outcome**	***n*** ** = 50**	**%**			***n*** ** = 50**	**%**				
Negative Outcome	46	92			14	28				
Moderate level of positive outcomes	4	8			31	62				
Positive outcomes	0	0			5	10			42.895	< 0.001
Mean ± SD	4.0 ± 2.0				8.4 ± 1.7				11.521	< 0.001

**P**: P value for **paired t-test** comparing between pre and post in studied patients. *: Statistically significant at *p* ≤ 0.05

Table 8: The study patients’ overall “satisfaction” scores pre-application of the Nursing Practices (42.1 ± 1.0) with a mean of 25.3 ± 1.8, whereas post-application of the Nursing Practice, it improved to (74.8 ± 4.9) with a mean of 83.9 ± 9.1. Significant differences were observed (P = < 0.001).

**Table 8:** Displays overall satisfaction scores of the studied patients pre and post applying the studied nurses` nursing practices

Overall satisfaction	Pre application of the Nursing Practices	Post application of the Nursing Practices	T	*P*
	Mean ± SD.	Mean ± SD.		
**Assurance**				
Total score	6.1 ± 1.5	10.7 ± 0.5	20.571	< 0.001
% score	25.7 ± 12.4	83.9 ± 5.7	30.155	< 0.001
**Empathy**				
Total score	5.9 ± 2.6	12.0 ± 1.8	38.013	< 0.001
% score	22.9 ± 9.9	70.0 ± 17.9	16.281	< 0.001
**Reliability**				
Total score	9.2 ± 2.4	15.4 ± 0.9	17.103	< 0.001
% score	26.7 ± 13.3	78.6 ± 7.6	23.957	< 0.001
**Responsiveness**				
Total score	5.3 ± 2.0	10.7 ± 0.5	18.521	< 0.001
% score	16.1 ± 7.1	83.9 ± 5.7	52.654	< 0.001
**Tangibility**				
Total score	19.6 ± 1.7	26.4 ± 2.9	14.303	< 0.001
% score	48.0 ± 8.2	82.0 ± 14.9	14.136	< 0.001
**Overall satisfaction**				
Total score	42.1 ± 1.0	74.8 ± 4.9	46.235	< 0.001
% score	25.3 ± 1.8	83.9 ± 9.1	44.669	< 0.001

**P**: P value for **paired t-test** comparing between pre and post in studied patients

*: Statistically significant at *p* ≤ 0.05

**Fig. 1:**
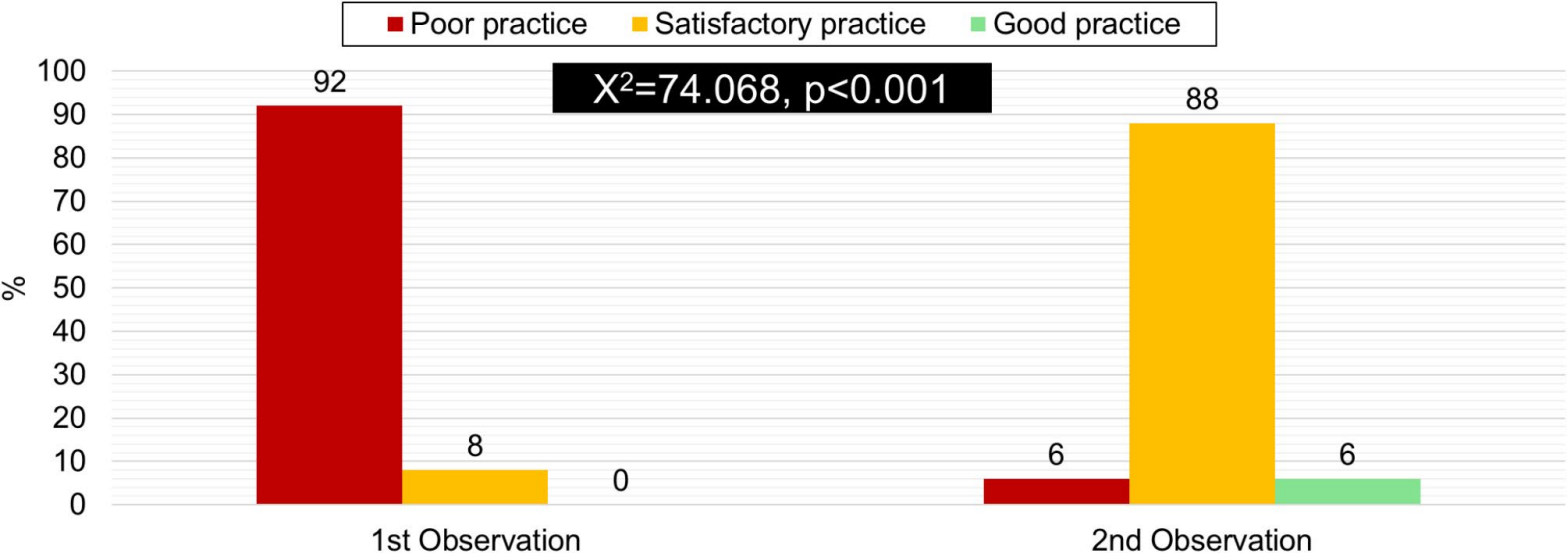
Comparison of the nurses total scores regarding the overall pre- and post-operative Nursing Practices between the 1st and 2nd observation periods

Figure 1 compares nurses’ total scores in nursing practices between the pre- and second observations pre- and post-applying NP. The findings demonstrated that 88% of the nurses studied gained a good practice score after implementing the Nursing Practices, whereas 92% had poor practice pre-implementing the Nursing Practices (NP). There is a statistically significant difference in overall scores for nursing practices between the first and second observation periods (P value < 0.001).

**Fig. 2:**
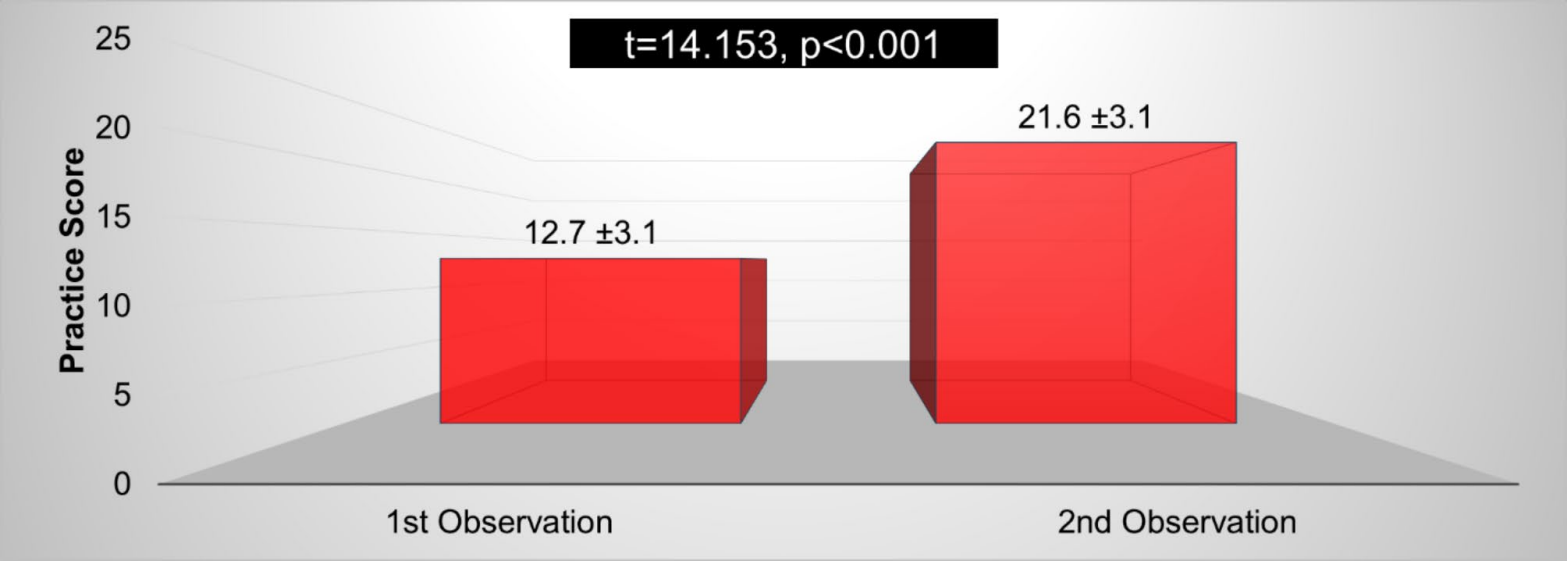
Comparison of the nurses’ total mean scores regarding the overall pre- and post-operative Nursing Practices between the 1st and 2nd observation periods

Figure 2 compares the nurse’s total mean scores in nursing practices between the pre- and second observations, pre- and post-applying NP, “which are one to two weeks”. The results revealed that the total mean score of nursing practices improved in the second observation (21.6 ± 3.1) compared to the first observation (12.7 ± 3.1). There is a statistically significant difference in total mean scores in Nursing practices between the first and second observation periods (P value < 0.001).

## Discussion

The present study aimed to evaluate the enhancement of nursing practice through patient outcome measures for optimizing care in intracranial surgeries. The results indicated a statistically significant improvement in nurses’ knowledge scores. Nurses demonstrated an enhanced understanding of pre-and postoperative precautions, seizure management, medication administration, infection control, and intracranial pressure management. These improvements align with the findings of Jasim [21], who reported that educational programs enhance nurses’ knowledge regarding craniotomy care. This increase in knowledge underscores the importance of structured educational initiatives like the NP in addressing gaps in specialized nursing knowledge.

Moreover, the current results are consistent with those reported by Kreem et al. [22], who noted a substantial improvement in nurses’ knowledge following an educational program on pre-and postoperative nursing management. However, these findings contrast with the study by Rongurliani [23], which indicated that nurses still needed to enhance their knowledge after a teaching program focused on craniotomy wound care. This discrepancy highlights the necessity of tailoring educational content to meet the specific needs of nurses in neurosurgical settings.

Conversely, another study by Soliman and Elhapashy [24] disagreed with the current findings, reporting that most participants achieved satisfactory knowledge levels in initial assessments due to continuous training and evaluation of nursing staff. This could be attributed to the positive impact of the Nursing Practices in enhancing nurses’ knowledge and fostering participant collaboration. Nonetheless, the current study’s findings contradict those of Kreem et al. [22], who identified a knowledge deficit among nurses concerning pre-and postoperative care.

Comparing pre- and post-intervention nursing practices, the study revealed that the Nursing Practices Standards (NP) significantly improved performance. The average practice scores increased compared to the first during the second observation period, with statistically significant differences observed. Most nurses demonstrated satisfactory practice levels during the second observation, particularly in headache assessment, patient education, medication administration, and history-taking. This study aligns with Abd El-Maksoud et al. [25] findings, which reported that most nurses adhered to infection control measures, managed seizures effectively, and provided pain management. However, there were deficiencies in wound care and neurological assessments.

Similarly, Pervaiz [26] found that nurses generally performed well monitoring vital signs after brain tumor surgeries. However, our findings contrast with those of Jasim [21], who discovered that more than two-thirds of nurses in Baghdad did not follow catheter management protocols post-craniotomy. While it was in contrast with Zhao et al. [27] Who mentioned that not only did the education/training enhance the nursing outcomes or patients’ self-care abilities and satisfaction, but also had a favorable effect on nurses’ practices, knowledge, and abilities. Feasibility and cost-effectiveness were the barriers to education and training, but management involvement and support, professional education and training, and the establishment of a regulated environment were the motivators.

Our study indicates that over two-thirds of the nurses achieved satisfactory practice levels during the second observation period. Nearly half performed adequately in wound care, pain management, infection control, and patient education on lifestyle modifications. These findings are consistent with those reported by Spears [28], who noted improvements in patient outcomes following educational interventions for nursing staff, and with Aiken et al. [29], who found that inadequate training often leads to adverse patient outcomes.

According to Horii et al. [30], traditional clinical training programs and self-reported competency assessments can help nurses enhance their technical knowledge and skills, including health education. However, one-quarter of the nurses still demonstrated poor practices, highlighting the need for ongoing education and support. From the investigator’s perspective, the improved practice levels can be attributed to the enhanced knowledge provided by NP AbdElhafiez et al. [31].

Regarding patient outcomes, the results showed that the study group had significantly better postoperative outcomes than the control group. Patients in the study group reported higher satisfaction levels following the implementation of NP. They perceived improvements in care quality across multiple dimensions, including assurance, empathy, reliability, responsiveness, and tangible outcomes. More than half of the patients reported positive lifestyle changes, improved headache relief, stable vital signs, and standard laboratory results. These findings align with those of Abd El-Maksoud et al. [25], who found that educational programs for nurses improved postoperative outcomes for patients undergoing brain surgeries. Similarly, Clement [32] reported that more than half of the nurses in their study achieved satisfactory knowledge levels, contributing to better patient care.

These results indicate that NP effectively enhanced nurses’ practices and patient outcomes. However, the findings differ from those of Rongurliani [23], who noted that nurses needed to improve their knowledge and skills regarding craniotomy wound care. In contrast, Abd El-Maksoud et al. [25] found that while nurses improved in wound care and infection prevention, many struggled with neurological assessments by Abboud [33].

The significant improvements observed in nursing practices and patient outcomes demonstrate the effectiveness of NP. Structured training programs and practice guidelines are essential to ensure high-quality nursing care for patients undergoing intracranial surgeries. The positive impact of NP underscores the importance of continuous professional development and education in enhancing nursing performance and patient outcomes. Hospitals should prioritize implementing similar initiatives to support nurses and promote better care for neurosurgical patients.

## Conclusion

Based on the current study’s findings, more than half of the nurses studied had satisfactory knowledge. In contrast, almost two-thirds had satisfactory practice levels regarding patients undergoing intracranial surgeries after implementing the nursing practices. Overall satisfaction scores of the patients studied were high. Patients’ overall “satisfaction” score pre-application of the Nursing Practices was (42.1 ± 1.0) with a mean of 25.3 ± 1.8, whereas post-application of the Nursing Practice, it improved to (74.8 ± 4.9) with a mean of 83.9 ± 9.1. Significant differences were observed (P = < 0.001). This study referred to the vital and valuable effect of good nursing practices on the quality of care for patients post-operatively and focused on the importance of nursing practice improvement based on continuous follow-up and training for nurses.

### Recommendation

It is essential to develop documented, up-to-date care protocols and handbooks to ensure that nurses have the necessary knowledge and can collaborate effectively while caring for patients who have undergone intracranial surgeries. The Nursing Practices checklist should be presented as a valuable tool for all nurses working in the neurological units at Mansoura University hospitals. Additionally, the role of liaison psychiatric nurses is crucial in alleviating patients’ anxiety before surgery and in monitoring and managing any mental health issues that may arise postoperatively. Adopt a randomized controlled trial design to strengthen causal conclusions. Increase sample size and select multiple hospitals to enhance generalizability. The integration of a follow-up period to evaluate long-term effects on patient outcomes and nursing practices is highly recommended.

### Limitation of the study

This study faced several limitations, including no follow-up period, which restricted the ability to assess the long-term effects of the Nursing Practices (NP) on patient outcomes and Nursing Practices. Reliance on self-reported data for nursing knowledge and practices may have introduced bias and could have influenced the implementation and outcomes. The quasi-experimental design lacked randomization, which limits causal inferences, and baseline differences between the study and control groups may have confounded results. Additionally, the study’s limited exploration of patient experiences beyond satisfaction scores leaves room for further research into patient perspectives on care.

### Nursing practices implications

The findings of this study highlight the critical role of nursing practices in improving outcomes for patients undergoing intracranial surgeries. Implementing Nursing Practices (NP) offers a structured, evidence-based approach to enhancing nursing knowledge and skills across preoperative, intraoperative, and postoperative phases of care. This can lead to improved patient recovery, reduced complications, and higher satisfaction. Nurses must be equipped with ongoing education and resources to apply NP effectively, emphasizing patient-centered care and proactive management of complications such as infections and intracranial pressure. Additionally, integrating outcome-based measures into routine nursing evaluations can ensure continuous quality improvement, ultimately fostering safer, more efficient, and patient-focused neurosurgical care. Integrating a follow-up period to evaluate long-term effects on patient outcomes and nursing practices is.

## Data Availability

No datasets were generated or analysed during the current study.

## Abbreviation

NP: Nursing Practices
